# Saltman on solidarity

**DOI:** 10.1186/s13584-015-0023-x

**Published:** 2015-06-10

**Authors:** Lawrence D. Brown, David P. Chinitz

**Affiliations:** Mailman School of Public Health Columbia University, New York, USA; Braun School of Public Health Hebrew University - Hadassah, Jerusalem, Israel

**Keywords:** Solidarity, Health reform, Economic austerity

## Abstract

Richard Saltman suggests that solidarity, a cherished notion at the heart of West European health care systems is being reconsidered in the light of today’s austere economic conditions. Solidarity, he argues, has always been a flexible moral guideline, one that allows for policy responses, such as limitations on health benefits or increased out of pocket payments, that challenging fiscal conditions are said to demand. Here we consider what the basic elements in solidarity – universality, redistribution, and uniformity-- mean in health as compared to other social policy realms such as pensions. Traditionally, the *commitment* to solidarity said little about the *contents* of services, but the latter is perhaps subject to increasing scrutiny under the health policy microscope. Saltman is right to emphasize the conceptual and cross-national flexibility of solidarity, but the notion retains a solid and durable core that continues to give valuable direction to policymakers in search of acceptable strategies and structures for decision making.

## Background

Richard Saltman suggests that solidarity, a cherished notion at the heart of West European health care systems is being reconsidered in the light of today’s austere economic conditions. Solidarity, he argues, has always been a flexible moral guideline, one that allows for policy responses, such as limitations on health benefits or increased out of pocket payments, that challenging fiscal conditions are said to demand. Here we consider what the basic elements in solidarity – universality, redistribution, and uniformity-- mean in health as compared to other social policy realms such as pensions. Traditionally, the *commitment* to solidarity said little about the *contents* of services, but the latter is perhaps subject to increasing scrutiny under the health policy microscope. Saltman is right to emphasize the conceptual and cross-national flexibility of solidarity, but the notion retains a solid and durable core that continues to give valuable direction to policymakers in search of acceptable strategies and structures for decision making.

## Commentary

Few would deny that ideological dogma is hard to banish from healthcare policy and the politics that shape it, but many may be surprised by Richard Saltman’s [1] contention that the sainted notion of solidarity is increasingly used to rule out, peremptorily and a priori, changes in health care coverage, benefits, and financing that aim to keep health care systems sustainable--no small task amid the hard, austerian times that now beset Western economies. Saltman argues skillfully that appeals to solidarity as an all-purpose rejoinder to attempts to constrain the growth of health care budgets is not only ill-advised but also ahistorical and myopic. In fact, he contends, societies purportedly devoted to solidarity have often departed from its presumed precepts, and in practice the content of the concept varies widely among societies and within societies over time.

Our comments here take issue less with his arguments than with his emphases: by treating solidarity mainly as a polemical encumbrance to grappling with the fiscal threats Western health care systems face, he obscures important parts of the policy picture. Moreover, the trio of nations he picks for discussion have three features—unusually long lags in embracing fully universal coverage, reliance on sickness funds for coverage, and a commitment to market forces (regulated competition) as a source of efficiency—that not only set them off from many Western peers (especially ones with single payer systems) but also entail distinct challenges to and complications in the definition and attainment of solidarity.

Contemplating the picture he sketches, one wonders whether solidarity is all variation and no (or very little) theme, whether there is any “there” there after all? The question is as much symbolic as it is ontological. Any health care policy, or change therein, may be examined from the philosophical high ground of justice or (and perhaps as) fairness and judged on criteria of equity and/or equality. What, if anything, does “solidarity” bring to the analytical party? Is it merely a political slogan, a rhetorical interloper, posing as a profundity but better suited to perplex than to guide policymakers? We contend that the term is most usefully approached not as a “criterion,” still less an “indicator” of good policy, but rather as an interpretive gloss on the goodness (justice, fairness, equity, and so on) of policy, what Blumer [2] called a “sensitizing concept.”

As Saltman points out, in the late 19th century solidarity meant a sense of fellow-feeling, of fraternity, which came to be extended into voluntary arrangements for mutual aid (e.g. the French “mutualites”). Solidarity initially connoted reciprocity among and on behalf of lower-earning workers, for whom illness could mean loss of jobs and incomes, a devastating outcome against which spreading risk and pooling resources offered protection. As the capacities of medical care grew and the costs of securing reliable care for a growing range of conditions rose, the scope of reciprocity expanded: “we” (meaning all citizens) are vulnerable, perishable, and in need of social support, but the smaller the pool of risks and resources, the harder this generalized reciprocity is to design and sustain. Voluntary arrangements could not achieve sufficient scale and durability so government became the vehicle of solidarity, and the scope of the “solidarity community” spread beyond the working classes to all citizens (a trend accelerated after World War II by the insistence of various international bodies that health care is a human right). In the years between the late 19th and the mid 20th centuries, then, the reach of solidarity as a normative guide to health care policy moved beyond the worker to the citizen and beyond the community to the nation.

Saltman seems to argue that, as a goal of and guide to health policy, solidarity is a concept with an infirm and shifting core, whereas we see it as a notion with a solid core within (in Wittgenstein’s term) “blurred edges.” Three ingredients constitute that core. The first is universality of coverage and access for all citizens and legal residents, though not necessarily those who can, or choose to, arrange private coverage (a rare exception, but one that appears in two or Saltman’s three vignettes, one being the Netherlands, which until recently exempted its top third of earners from the national health insurance system, and the other Germany, which continues to exempt the highest earners and civil servants from its system of NHI). The second element is redistribution: intrinsic to solidarity are cross-subsidies from the better off, the healthier, the younger (and so on) to the poorer, the sicker, and the aged. The third basic feature is uniformity: everyone in the system of coverage should be able to access the system of care on more or less equal terms, which means in practice that care should be allocated solely on the basis of medical need. (In Germany, for example, the concept of solidarity as codified in law entails the pooling of contributions and expenses without regard to individual or sex-specific risk assessments; means-tested contributions by which better off participants support the less well off; and support for participants with dependents by those who have none [3]).

Health care is not, of course, the sole venue in the welfare state in which solidarity looms large. In France, for example, “social security” contains other regimes—retirement benefits (pensions), family allowances, unemployment benefits, and benefits for occupational injuries--besides health care, all of which are expected to honor solidarity. But the application of this “principle” to (say) pensions is much more straightforward than is the case for health care. Universality means, in essence, that all workers who had retirement contributions extracted from their paychecks over their career get benefits. Redistribution is achieved by giving some retirees benefits of substantially greater monetary value than the contributions they made. And the terms, timing, and amount of benefits are spelled out uniformly by law. Issues can and do arise: should the best off receive payments they do not really “need”? Should benefits be larger or smaller, awarded earlier or later? These decisions are made and revised differently among nations and within nations over time. But it is not especially hard to identify and explicate the solidaristic core of pension policy.

Health care policy poses distinctly different challenges. In this case, universality means not that a check is reliably in the mail but that all citizens can get the various medical services they may need over the course of their lives as needs arise. Solidarity insists that no citizens should have trouble accessing the system (or affording the services they received) for financial or other reasons unrelated to need. Universality of coverage follows from universal vulnerability to illness. (Even in the US, the most obdurate opponents of universal coverage insist that those who lack coverage can still get care, backhandedly conceding the moral claim of universality.) In pension policy beneficiaries are entitled to a sum of money; in health care they are entitled to enter the multiple systems (financing, delivery, and so on) that define how health services play out in distinct clinical circumstances.

Solidarity as universalism is not a strategic straight jacket: for example, cost sharing and copayments, cannot be condemned a priori as unsolidaristic. These measures tend to be accompanied by exemptions—for the disabled, the aged, children, pregnant women, veterans, and others—that redistribute their impact. Citizens pay for their health care coverage and care in taxes (payroll, income, sales, and other) and by trading off increases in wages and salaries, and solidarity demands that these payments be calibrated to ability to pay. Absent more detail (for example, about the groups for which cost sharing charges are waived or reduced) and more context (in nations in strained financial circumstances perhaps sizable cost sharing is required to sustain basic health coverage and care for all citizens) one cannot judge whether, and how far, eye-popping figures (for example, the 37.4% cost sharing that Saltman cites in Latvia) offend solidarity [4].

As for redistribution, solidarity spotlights the proposition that coverage and access can be universal only if cross-subsidies flow from the more advantaged to the less advantaged. This is what equity entails, indeed denotes-- at least in Europe. (In the US a sizable body of opinion seems to believe the reverse, namely, that good health risks deserve to get good health insurance rates.) Those transfers sustain coverage (that is, entry into the system), but the contents of coverage (what is in the benefit package and what providers deliver) is another matter. With pensions, everyone gets a floor and the resources they enjoy beyond that floor depend on lifetime earnings, investment strategies, spending habits, and so forth. In health care too everyone gets a floor—access to medically necessary and appropriate care—but the construction of the floor differs with clinical need as defined by medical providers. Traditionally, the *commitment* to solidarity has said little or nothing about the *contents* of services, and the type of adjustments in the coverage or provision of one or another service that Saltman describes usually have been relatively obscure or carried out implicitly and thus have not been felt as a challenge to or a “reduction” [1] of solidarity. Solidarity, then, has been understood not as a mandate for any particular set of benefits or services but rather as systematic cross-subsidization of entry into a system in which what is medically necessary and appropriate remains to be determined. Increasingly, however, concerned observers contend that some systems are seeking to set explicit limits to health benefits in ways that sacrifice solidarity for cost containment.

In pension policy uniformity is easy: everyone meeting simple criteria (age, contribution, and so on) gets paid. Not so health care: everyone gets (or can get) care, but not everyone can expect to get every possibly beneficial clinical service. Systems of universal coverage have always recognized the need for limits, which need, of course, grows only more salient under the pressure of relentless medical innovation. In France, this recognition long ago led to the “ticket moderateur,” (copayments intended to “moderate” consumption of care) and in England to gatekeeping (the requirement that patients must see their general practitioner before visiting a specialist). Because services that threaten the financial sustainability of the system are not generalizable (that is, appropriate to be uniformly on offer), one should be cautious about characterizing denial of or limitations in coverage of such services as reducing solidarity with one or another group.

Is this type of rationing inherently antisolidaristic? Some nations—for example, England and Israel—say no explicitly and unapologetically. All systems have procedures for assessing new technologies, and solidarity is usually a central and explicit consideration in their decisions. (On the importance of solidarity in setting priorities in Germany’s Institute of Quality and Efficiency in Health Care [IQWIG] see Katharina Kieslich [5]). But how decisions are reached and enforced varies with rules governing coverage and payment, demands and preferences of consumers, judgments by providers, supply side considerations (number of specialists, beds, and so on), and availability of alternatives (for instance, palliative and hospice care instead of protracted hospital stays). Societies work out these matters (which is to say, their practical understanding of the scope and limits of solidarity) for themselves. Some nations—for instance, The Netherlands and Sweden—have held elaborate ethical inquiries into how to define the scope and limits of “the solidarity community” for health care services. The upshot is that most services traditionally and currently covered pass muster, and that there is a limit to the time and energy commissions are willing to spend debating whether in vitro fertilization or Viagra respond to “social” or “merely personal” needs. All the same the relentless pace of medical innovation increasingly forces pointed and poignant decisions about whether to exclude new technologies with limited benefit according to experts but “life saving” qualities in the eyes of patient groups and the media. Such processes could fatally corrode solidarity, but evidence from Israel indicates that meticulous and consultative decision making by expert bodies can reduce the risk of decisions that fail to give solidarity its due [6].

Western systems, moreover, demarcate a special and separate set of services that lie beyond the realm of (what they take to be) medically necessary and appropriate care and maintain complementary, supplementary, or private coverage regimes that, for example, allow a patient faster access to a specialist (England), reimburse a patient for some copayments (France), and pick up the cost of dental and eye care (pretty much everywhere). In general these systems seem to accept that a flexible ceiling on services is consistent with solidarity so long as the floor (access of all to medically necessary and appropriate services) is secure, but in many, the demands of solidarity are a topic of lively debate. For example, Canadians have long frowned on enabling citizens to buy private coverage that permits them to jump the queue for care, but they may be changing their collective (anyway judicial) mind on the matter. Worried that less advantaged citizens had trouble affording the complementary coverage enjoyed by the better off, France in 2000 made complementary coverage universal and now debates how best to limit the financial burdens of eye care, dental services, and prosthetics for citizens on public assistance [7]. Moreover, troubled that the state’s heavier reliance on the complementary sector may have disparate effects across the full range of insured citizens, France is considering whether public regulation should introduce more uniformity into a sector in which market forces have heretofore enjoyed fairly free play.

In Israel, supplementary insurance is subject to guaranteed issue requirements, is community rated by age, and is held by nearly 80 % of the population. Its use by some subscribers to expand their choice of physician, and to jump the queue for consultations and surgeries in private or quasi private settings, however, has challenged solidarity. Proposals to expand this option to the nation’s public hospitals are resisted by both the Israeli Ministry of Finance (which fears an increase in spending in the public hospital sector) and a recent blue ribbon panel (which decried it as inequitable because supplementary coverage, while widespread, is not universal).

In these cases one sees both the durability of the core of solidarity and the productive “dialogical” role it plays in health policy deliberations: all should be covered for medically necessary and appropriate care, but the boundaries of such care are porous and sometimes contentious, so considerations of solidarity (universality, redistribution, and uniformity) should shape the rules of the game that govern complementary coverage too. Keeping solidarity in play does not chart detailed policy paths but rather serves to italicize crucial questions, to refine policymakers’ reflections on them, and to identify policy making processes and institutional arrangements that can make and implement decisions about the evolving roles of complementary insurance.

Saltman skillfully sketches the gains and losses in solidarity that have followed the move to regulated competition in the Netherlands in 2006, but he says little about an important contextual part of the picture. The 20-odd years between the arrival of regulated competition on the Dutch health policy agenda in the mid 1980s and its enactment in 2006 saw not only the search for a supportive political coalition but also endless tinkering with a risk adjustment formula for paying health insurers, in order to forestall the preferred risk selection that competition, absent such a feature, might unleash. The formula was deemed crucial to ensuring that competitive efficiencies did not come at the cost of solidarity, which cannot abide the barriers that experience rating throws in the path of universal coverage. The ups and downs of solidarity that Saltman emphasizes, in short, unfolded within a larger policy framework within which the protection of solidarity was accepted by virtually all policy protagonists as a top priority.

It is worth noting too that in most Western nations, the challenges to solidarity that accompany estimations of risk, adjustments of payments to insurers to account for risks and tensions over limited provider networks do not arise. Saltman’s vignettes feature three of the four main Western national experiments (Switzerland is number four) that seek efficiencies by encouraging competition among sickness funds. Single payer systems (Canada, England, Italy, Spain, and the Scandinavian nations, for example) do not use sickness funds for basic coverage, and some nations that do use them (France, for instance) resist the seductions of market competition in health coverage. Policymakers in these societies are not unaware of the theoretical claims on behalf of competition, but they resist them for many reasons, not the least important being that it could damage if not destroy solidarity.

Solidarity need not mean a principled disregard of the risk profile of the citizens a universal system covers, however. Germany, convinced that this concept entails duties as well as rights, now permits sickness funds to offer incentives for participation by their enrollees in wellness programs, the rationale being that solidarity obliges citizens to avoid needless illness and use of care that wastes the system’s scarce resources [3]. In this case, too, solidarity serves as an invitation to dialogue: do these particularized incentives breach solidarity by introducing invidious distinctions among citizens and by adding behavioral incentives to the calculation of their contributions to the cost of their coverage, or do they enhance it by helping to shore up a collective good, namely, collective coverage? The question is debatable—and perhaps unanswerable—but the importance of collective endeavors to explicate it is undeniable.

The slow economic growth that has followed the Great Recession of 2008 certainly portends “austerity” and perhaps even “permanent retrenchment” for Western health care systems, but these systems have been wrestling with challenges comparable in kind if not in degree for decades. Cut backs have mainly meant marginally slower growth in health care spending which, as noted above, cannot be said per se to violate solidarity, because the verdict depends on what happens to whom as the growth of health care spending slows. In single payer systems, the main manifestation of these “cuts” has been longer wait times to enter the system, and one would need an accurate picture of their incidence across groups and regions in order to gauge their effects on solidarity. In Continental systems, proponents of solidarity among policy elites in the public sector have since the 1980s augmented their expertise and refined their arguments in favor of a tenacious and strong state presence—a position they have defended by incorporating new fiscal constraints into their public policy agenda. In France, for instance, elites within Social Security agencies “used the argument of budgetary constraint to [their] advantage and ensured the durability of the French welfare model, that is, a sustainable Social Security.” In other nations too, a strategic fusion of solidarity and sustainability lets defenders of a strong state articulate “plausible public claims about helping democracies adapt to changing economic and international environments” [8]. Saltman rightly remarks that “past practice clearly indicates that governments can and regularly do change the social contract that underlies health sector solidarity” and that further changes will not necessarily reflect a breach of stewardship [1]. Nevertheless European nations continue to look much more like each other than they look like the United States, where policymakers shun the term “solidarity” in principle, admit it into practice solely for the aged and for veterans, and (notwithstanding some steps toward standardization under the Affordable Care Act of 2009) largely entrust considerations of universality, redistribution, and uniformity to the whims of employers and the 50 states. The difference is that in Europe, unlike the US, the variations on which Saltman chooses to focus play out around a firm and (so far) stable conceptual and normative core of solidarity which pervades not only the substance of health policy, in particular the cultural resolve that all citizens should get the care that they need without financial barriers, but also the processes created to determine the limits to what patients and citizens can expect to be able to access.

Many of the best technical tools for making such decisions – for instance, health technology assessment, cost-effectiveness analysis, and evidence based medicine – have US origins, but absent firm underpinnings of solidarity their effects have been limited, and sometimes, by triggering hysteria over alleged rationing of care by federal bureaucrats, even perverse . Allegiance and attention to the core value of solidarity, and the commitment to explore its practical meaning in shifting strategic contexts enable European systems to apply these tools in ways that are (or anyway aim to be) both principled and pragmatic.

## Conclusion

Saltman’s caution that shrill voices on the Left should not be allowed to invoke solidarity as an all purpose antidote to efficiency and sober-minded budgeting is useful. We would think it unfortunate, however, if his caveats fortified forces on the Right who would dismiss solidarity as a metaphysical chimera and a luxury Western health care systems can no longer afford.

## References

[1] Saltman R (2015). Health sector solidarity: a core European value but with broadly varying content. Isr J Health Policy Res.

[2] Blumer H (1954). What is Wrong with Social Theory?. Am Sociol Rev.

[3] Elliott H, Bernstein J, Bowman DM (2014). Wellness as a Worldwide Phenomenon?. J Health Polit Policy Law.

[4] Tambor M Milena Pavlova M Rechel B, Golinowska S, Christoph Sowada C, Wim Groot W (2014) Eur J Public Health 24 (3): 378–38. 10.1093/eurpub/ckt118 PMC4032479 24065370

[5] Kieslich K (2012). Social values and health priority setting in Germany. J Health Organ Manag.

[6] Chinitz D, Meislin R, Grau I (2009). Values, Institutions and Shifting Policy Paradigms. Health Policy.

[7] Tournaine M (2014). Intervention : Presentation de projet de loi santé, 13octobre 2014.

[8] Genieys W, Hassenteufel P (2015). The Shaping of New State Elites: Healthcare Policymaking in France Since 1981. Comparative Politics.

